# Focus Issue on Male Infertility

**DOI:** 10.1155/2012/823582

**Published:** 2011-12-01

**Authors:** Hideyuki Kobayashi, Koichi Nagao, Koichi Nakajima

**Affiliations:** Department of Urology, Toho University School of Medicine, Tokyo 143-8541, Japan

## Abstract

Male infertility problems can occur when sperms are limited in number or function. In this paper, we describe the clinical evaluation of male infertility. A detailed history, physical examination, and basic semen analysis are required. In addition, ultrasound, karyotyping, and hormonal studies are needed to determine specific causes of infertility. In addition, the World Health Organization (WHO, 2009) has developed a manual to provide guidance in performing a comprehensive semen analysis. Among the possible reasons for male infertility, nonobstructive azoospermia is the least treatable, because few or no mature sperm may be produced. In many cases, men with nonobstructive azoospermia typically have small-volume testes and elevated FSH. Although treatment may not completely restore the quality of semen from men with subnormal fertility, in some cases a successful pregnancy can still be achieved through assisted reproductive technology.

## 1. Introduction

About 1 in 7 couples have problems conceiving, with a similar incidence worldwide. Over 80% of couples who have regular sexual intercourse and do not use contraception will achieve a pregnancy within one year, and approximately 92% can achieve a pregnancy within 2 years [1]. Infertility affects males and females equally, although many people believe that infertility is a female problem. In Japan, especially, couples oppose insemination or adoption as an alternative to having a child carrying both parents' genes, which means that males are likely to seek infertility evaluations when a couple has difficulty conceiving.

 The clinical evaluation of male infertility includes a detailed history, physical examination, laboratory tests, ultrasound study, and karyotyping. The two main purposes of the evaluation are (1) to identify any modifiable factors that can improve the man's fertility status and (2) to identify any serious underlying conditions, such as testis cancer, osteoporosis, and endocrine or genetic problems that present first as infertility [2].

## 2. History-Taking for the Male Infertility Workup

The infertility history should include a detailed account of the patient's reproductive and sexual history, developmental, family, medical, and surgical history. The information to be included in each portion of the history is detailed below.

### 2.1. Reproductive and Sexual History

For the reproductive history, any prior conceptions for the male with present or past partners, details of any prior difficulty achieving conception, past evaluations and treatments for infertility, and previous use of contraception should all be recorded, along with the frequency and timing of intercourse with the man's current partner. Information about erectile and ejaculatory function and frequency of masturbation should be requested, as well as the timing of first masturbation and intercourse.

### 2.2. Developmental History

A history of specific childhood illness or conditions may be informative. For example, bilateral cryptorchidism causes a significant decrease in spermatogenesis, but unilateral cryptorchidism usually has much less impact. Studies of patients who underwent orchiopexy to treat cryptorchidism report decreased sperm densities in about 30% of men, on average, with unilateral cryptorchidism (range 28–82%), although two studies have reported abnormal sperm densities in only 17% of patients [3, 4]. In contrast, an average of about 50% of patients with bilateral cryptorchidism (range 9–88%) show decreased sperm densities. Despite the trend of performing orchidopexies at an earlier age, improved fertility rates have yet to be demonstrated with this approach. On the other hand, testes that remain undescended after puberty do not function, and fertility rates are not improved by postpubertal repair [5, 6].

Testicular trauma or a history of torsion should be noted, since both may result in atrophic testes. Approximately 30–40% of men with a history of testicular torsion have abnormal results upon semen analysis [7–14]. In up to 11% of patients with testicular torsion, antisperm antibodies are present at the time of or after the event [15, 16].

The timing of pubertal development should be noted. Significantly delayed or incomplete development may suggest an endocrinopathy. In addition, although early childhood mumps does not appear to affect the testis, after the age of 11 or 12, 30% of male patients who contract mumps develop unilateral orchitis. Bilateral orchitis occurs in approximately 10% of peripubertal and adult males who contract mumps [17]. Unilateral and bilateral orchitis from mumps can cause severe testicular damage.

### 2.3. Medical History

Diabetes may affect erectile and/or ejaculatory function [18], and any systemic illness accompanied by fever or viremia can lead to impaired testicular function, although the effects may not be measurable in the ejaculate for 1–4 months. A history of pyospermia or prostatitis should be noted, although both are uncommon and neither is proved to cause infertility [19]. Primary ciliary dyskinesia (also known as immotile cilia syndrome), which should be suspected when there is a history of chronic upper respiratory infections, causes severe defects in sperm motility. When this condition is associated with situs inversus, it is known as Kartagener syndrome, which is a rare cause of male infertility [20]. Frequent respiratory infections associated with azoospermia raise the possibility of Young syndrome [21], in which epididymal obstruction is caused by the inspissation of secretions. Neurologic issues can lead to male infertility as a variety of hormonal abnormalities including thyroid disorders, hyperprolactinemia, and elevated estrogen levels. Finally, any history of urinary tract infections or sexually transmitted disease should be recorded, particularly if associated with epididymitis, as these conditions can lead to epididymal obstruction.

### 2.4. Past Surgical History and Cancer Treatments

Details of past surgeries should be obtained. Pelvic and retroperitoneal surgery may impair ejaculatory function.

Patients with testicular cancer may present with infertility either before or after treatment. Approximately, 50% of testicular cancer patients have subnormal sperm densities prior to chemotherapy [11, 13, 22, 23]. Following cisplatinum-based chemotherapy for testicular cancer, most patients will develop azoospermia. However, most will recover sperm production within four years [24, 25]. Most patients with lymphoma, leukemia, or sarcoma become permanent sterility after chemotherapy.

### 2.5. Medication and Drug Use

A detailed history of medications should be obtained. Unfortunately, for many substances, data detailing potential effects on male fertility are lacking. Online sources include databases such as Reprotox and Reprotext. Both are often available through online hospital and university databases. Exogenous androgens are well known to induce hypogonadotropic hypogonadism, which may be induced directly by testosterone or by synthetic anabolic steroids. The subsequent suppression of endogenous testosterone production usually results in azoospermia, which is frequently reversible over a 3–6-month period. Importantly, some patients do not recover normal pituitary function.

### 2.6. Lifestyle Exposure

Exposure to potential environmental toxins including pesticides, mercury, cadmium, arsenic, and hydrocarbons can impair spermatogenesis. In particular, the soil fumigants amebicide and nematocide, and 2-bromopropane (a substitute for chlorofluorocarbons) can cause decreased spermatogenesis [26–32]. Alcohol dependency is frequently associated with testicular atrophy, but moderate alcohol consumption does not appear to impair fertility [33–36]. The effect of cigarette smoking on male infertility remains controversial, with some studies showing decreased semen health and others showing no effect [37–43]. However, the sum of the accumulating evidence suggests that cigarettes smoking can impact male fertility.

## 3. Physical Examination

The physical examination for male infertility should focus on identifying abnormalities that could affect fertility. Endocrine disorders should be suspected in cases of abnormal androgenization. Gynecomastia can result from excessive estrogens, an improper estrogen-to-androgen ratio, or elevated prolactin levels. Penile curvature, angulation, and the location of the urethral meatus should be assessed. The scrotum should be carefully palpated with the patient standing, noting the size and consistency of the testicles; the room should be kept warm for this exam.

 The testicle size can be measured with an orchidometer. This measurement is important because impaired spermatogenesis often accompanies small-volume testes [44]. The normal volume is at least 20 mL [45, 46]. Note, however, that Asian men typically have smaller testes than men of other races. The epididymis should be examined carefully, noting the presence of the caput, corpus, and cauda as well as whether the epididymis feels full or indurated. Fullness can suggest obstruction of the genital ducts.

During palpation of the spermatic cords, the clinician should confirm the presence or absence of the vas deferens and note any vasal atrophy or nodularity, or the presence of a varicocele within the spermatic cord and surrounding the testicle. An enlarged venous diameter can be detected with the patient performing the Valsalva maneuver. Alternatively, the patient can often increase intra-abdominal pressure without contracting the cremaster muscles, by distending the abdomen. Varicoceles are graded as small (grade I) if palpable only when the patient performs the Valsalva maneuver, moderate (grade II) if palpable when the patient is standing but does not perform the Valsalva maneuver, and large (grade III) when veins can be seen through the scrotal skin. Varicoceles should decrease in size when the patient is in the supine position.

## 4. Laboratory Tests

After the above exams have been completed, appropriate laboratory tests should be performed. The first step in laboratory testing is to identify patients who are likely to be infertile, subfertile, and fertile, for which only semen analysis is required. Since most infertile men have some motile sperm in the semen, threshold values are used to indicate whether fertility is more or less likely. Azoospermic patients, however, are sterile. Importantly, although the number of motile sperm is suggestive of fertility, there can be considerable variation in the fertility of men with equal motile sperm counts.

### 4.1. Semen Analysis

The World Health Organization (WHO, 2009) has published a guide to semen analysis in humans. Because semen parameters fluctuate from day to day, at least two semen samples are usually required to diagnose below-normal semen quality. A recent study of over 4500 samples from men living in four continents was used to determine the reference range for adults. The lower reference limits (5th percentile) for semen parameters in “fertile” adult men are given in Table 1 [47].

#### 4.1.1. Collection

Patients should be given specific instructions on collecting semen specimens for analysis. Semen is collected by masturbation. Patients should be encouraged to abstain from ejaculation for 72 hours prior to collecting a semen sample in the laboratory. We provide adult videos and books for patients in a room dedicated to semen collection. The specimen container should be clean, although sterility is not required, and wide-mouthed, to minimize collection error. The specimen should be transported to the laboratory at room temperature. Note that fresh semen is a coagulum that liquefies 5–25 min after ejaculation.

#### 4.1.2. Concentration

The term, “sperm count,” typically refers to sperm density, reported as millions of sperm per milliliter of semen. “Total sperm count” refers to the total number of sperm in the ejaculate.

### 4.2. Interpretation of the Initial Evaluation

Once the data from the physical examination, history, and semen analysis have been acquired, a differential diagnosis should be developed. Additional laboratory studies may then be ordered to refine the diagnosis and determine management options. The results from the semen analysis are categorized as all parameters normal, oligozoospermia, asthenospermia (defects in motility), teratozoospermia (defects in morphology), and azoospermia (complete lack of sperm).

#### 4.2.1. Interpretation of Normal Semen Parameters

A finding of normal semen parameters in the context of infertility raises the possibility of a female factor, erectile dysfunction, and/or antisperm antibodies.

## 5. Diagnosis

### 5.1. Differential Diagnosis of Azoospermia

Azoospermia is categorized as either obstructive or nonobstructive. Nonobstructive azoospermia refers to a lack of sperm production, whereas obstructive azoospermia implies adequate sperm production but failure to deliver the sperm into the ejaculate because of a ductal obstruction. The data on the testicular size and presence of the vas deferens relate to this diagnosis, and congenital bilateral absence of the vas deferens (CBAVD), an obstructive azoospermia, is diagnosed by physical examination.

 Atrophic testes may be primary, due to an inherent testicular dysfunction, or secondary, due to a hormonal deficiency. Azoospermic patients with normal semen volumes and a palpable vas deferens should have their FSH and testosterone levels measured. The finding of small testes in association with low FSH and testosterone suggests hypogonadotropic hypogonadism, and the patient's LH and prolactin levels should be evaluated as well. Low gonadotropin levels associated with elevated prolactin raise the possibility of a pituitary prolactinoma, and a pituitary MRI should be performed.

The finding of atrophic testes and elevated FSH levels indicates germ cell failure. Patients with normal sperm production typically have FSH values in the lower end of the normal range, and levels above this should raise suspicion of a defect in spermatogenesis. In addition, patients with unilateral testicular disease may have elevated FSH levels. A diagnostic testicular biopsy is not indicated in patients with elevated FSH levels. Instead, patients with nonobstructive azoospermia due to a primary testicular defect and not to a hormonal deficiency should be offered genetic testing, consisting of a karyotype and a Y-chromosome microdeletion analysis. If abnormalities are found, a couple should be offered genetic counseling prior to proceeding with assisted reproductive techniques.

 Normal-sized testes accompanied by normal FSH and azoospermia suggest the possibility of obstruction, and a testicular biopsy is required to differentiate between obstruction and maturation arrest. If mature sperms are found in the biopsy, sperm may be cryopreserved for later use in an IVF/ICSI cycle.

 Low-volume azoospermic semen specimens may be caused by hypogonadism (low testosterone levels), ejaculatory duct obstruction, or seminal vesicle absence or hypofunction. Patients with bilateral ejaculatory duct obstruction and those with congenital bilateral absence of the vas deferens (CBAVD) produce low-volume, acidic, and azoospermic semen specimens.

### 5.2. Multiple Semen Abnormalities

The finding of defects in sperm density, motility, and morphology is commonly referred to as oligoasthenoteratozoospermia (OAT). By far the most common cause of this pattern is varicocele, which is a clinical diagnosis determined by a physical examination and ultrasound study. Additional causes of OAT include environmental toxins, drugs or medications, and cryptorchidism.

### 5.3. Defects in Isolated Semen Parameters

Complete ejaculatory failure, also called aspermia, exists when no fluid is produced during the male orgasm, and it is due to retrograde ejaculation. Common causes of ejaculatory failure include neurogenic abnormalities, such as spinal cord injury, diabetes mellitus, multiple sclerosis, and the use of *α*-blockers. Retroperitoneal surgeries, including pelvic surgery and retroperitoneal lymph node dissections, can also result in impaired ejaculation. True absence of ejaculation should be differentiated from the complete inability to obtain orgasm, which can be caused by medications such as serotonin reuptake inhibitors and/or by psychological disturbances. However, much more common than complete lack of ejaculation are low-volume ejaculate specimens. The most common cause of this finding is an incomplete collection. Complete bilateral ejaculatory duct obstruction will result in azoospermic, low-volume, and acidic semen specimens.

### 5.4. Defects in Sperm Concentration

In patients with less than 10 × 10^6^ sperm/mL, serum FSH and testosterone levels should be determined. In patients with less than 5 × 10^6^ sperm/mL, a karyotype and Y-chromosome microdeletion analysis should be considered as well. Elevated serum FSH may indicate a primary testicular defect. Although varicoceles are a common cause of low sperm density, other abnormal sperm parameters are also usually present with varicocele.

### 5.5. Defects in Motility

The term “asthenospermia” refers to defects in sperm movement. Such cases show only a low percentage of sperm that have any motility. Antisperm antibodies and varicoceles may account for this defect.

### 5.6. Defects in Morphology

The term “teratozoospermia” refers to defects in sperm morphology. This has been reported with the application of “strict” criteria. The majority of the cases are idiopathic, while varicoceles and temperature insults to spermatogenesis are also potential causes.

## 6. Additional Laboratory Tests

### 6.1. Hormonal Studies

Hormonal evaluation for male subfertility is done to (1) identify causative endocrine abnormalities or (2) obtain prognostic information. Endocrine studies should include serum levels of PRL, LH, FSH, testosterone, and E2 (estradiol). As mentioned above, the most common endocrine abnormality associated with male infertility or subfertility is elevated FSH, which generally indicates the impairment of spermatogenesis. However, FSH levels may be normal in cases of impaired spermatogenesis.

 True endocrine causes of male infertility account for less than 3% of cases [48].

### 6.2. Antisperm Antibodies

Immunologic infertility is an autoimmune disorder that can involve both the humoral and cellular immune system. However, the mechanism is still poorly understood. Note that other causes may account for cases of male infertility, even when antisperm antibodies are detected. Treatment of antisperm antibodies is the use of ICSI to bypass potential fertilization problems induced by the antibodies.

## 7. Conclusions

While many assays can be used in the diagnosis of male infertility, the foundation of any workup should be obtaining a detailed history, thorough physical examination, and a basic semen analysis. Additional tests, including an antisperm antibody assay and hormonal, karyotype, and ultrasound studies are needed only to investigate specific causes of infertility. In addition, there are some significant health issues which are diagnosed only with presentation of male infertility, testis tumor and significant endocrinopathies amongst them. A careful evaluation that considers all of the possible explanations for a man's infertility is not only critical for a good diagnosis, it is an ethical imperative for the reproductive specialist.

## Figures and Tables

**Table 1:** Semen parameters.

Volume	>1.5 mL
Sperm concentration	>15 million/mL
Total sperm count per ejaculate	39 million
Sperm progressive motility	>32%
Total sperm motility	>40%
Sperm vitality	>58% alive
Normal morphology	>3%
WBC (white blood cells)	<1 million/mL
